# Progress of Gastric Cancer Surgery in the era of Precision Medicine

**DOI:** 10.7150/ijbs.56735

**Published:** 2021-03-02

**Authors:** Yumin Wang, Luyuan Zhang, Yi Yang, Shan Lu, Hao Chen

**Affiliations:** 1 Department of General Surgery, The First Affiliated Hospital of USTC; Division of Life Sciences and Medicine, University of Science and Technology of China, Hefei, Anhui, China.; 2 Department of Otolaryngology Head and Neck Surgery, Xiangya Hospital, Central South University, Changsha, Hunan, China.; 3 Department of Neurosurgery, First Affiliated Hospital, School of Medicine, Zhejiang University, Hangzhou, China.; 4 Department of Nuclear Medicine, Xiangya Hospital, Central South University, Changsha, Hunan, China.; 5 Department of Radiation Oncology, Harbin Medical University Cancer Hospital, Harbin, Heilongjiang, China.

**Keywords:** gastric cancer, surgery, precision medicine, chemotherapy, immunotherapy

## Abstract

With the development of genomics, the update of modern imaging technology and the advent of artificial intelligence and big data, the surgical treatment of gastric cancer has gradually stepped into precision medicine. Precision surgery treatment of gastric cancer is based on accurate molecular typing and staging using modern molecular diagnostic technology and imaging, and the formulation of precise and individualized surgical treatment plans, with the concept of minimally invasive and accelerated rehabilitation surgery running through it. For intermediate-stage gastric cancer, we have adopted a comprehensive treatment approach including traditional radiotherapy and chemotherapy, targeted therapy and immunotherapy. Utilize artificial intelligence and big data technology to improve the standardization and interconnectivity of specialty data and realize the transformation of evidence-based medicine. Promoting the standardization, standardization and individualization of gastric cancer surgical treatment, providing patients with precise diagnosis and treatment, and further improving patients' prognosis are the opportunities and challenges in the development of gastric cancer surgery.

## Introduction

Gastric cancer ranks second among all malignant tumors in terms of morbidity and mortality globally 1, posing a great threat to people's health. Surgery is currently the only means to cure it 2. With the initial completion of the Human Genome Project and the widespread application of next-generation sequencing technology, human research on the pathogenesis of malignant tumors has made great progress 3. Artificial intelligence has made revolutionary progress in multiple medical scenarios such as disease diagnosis, drug screening, imaging medicine, and nursing medicine. Pathological slices are the primary breakthrough point of artificial intelligence, the standardization and digitization of pathological slices provide a big data background for the deep learning of artificial intelligence. The new medical model developed at the intersection of sequencing technology and big data science aims to guide targeted therapy through genomics, proteomics and other technologies, and ultimately achieve the goal of individualized and precise treatment of diseases 4. In the future, the concept of precision medicine will run through the whole process of malignant tumor prevention, diagnosis and treatment. In the field of gastric cancer surgery, with the development of modern imaging and the innovation of minimally invasive technology, the surgery is gradually developing in the direction of minimally invasive and precise 5. In this review, we will discuss the current status and progress of gastric cancer surgery in the era of precision medicine in terms of gastric cancer classification, preoperative evaluation, surgical methods, perioperative neoadjuvant therapy and artificial intelligence.

## Changes of gastric cancer classification

**Figure 1:**
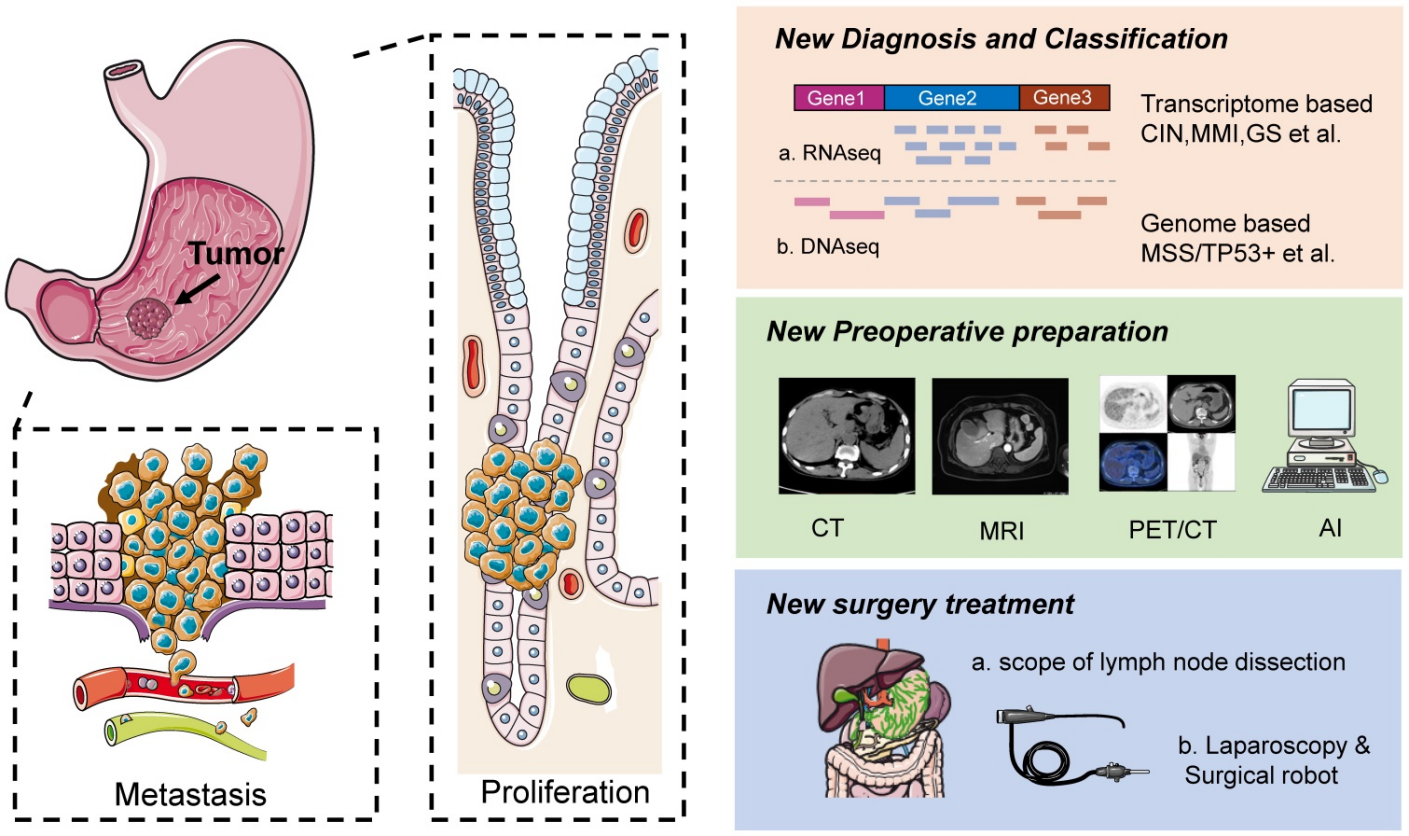
** Occurrence, diagnosis and treatment of gastric cancer.** Left is schematic diagrams of the occurrence, development, invasion and metastasis of gastric cancer. Right is schematic diagrams of the diagnosis, preoperative preparation and treatment of gastric cancer.

Gastric cancer can be classified into Borrmann's classification and Siewert's classification according to anatomical origin, WHO classification and Lauren's classification according to histological structure, and early stage and late stage according to disease severity 6. In recent years, with the rapid development of cancer genomics and transcriptomics, cancer is classified into “molecular” subtypes, which theoretically better reflect the biological behavior of gastric cancer 7 **(Figure 1).**

### Transcriptome based gastric cancer classification

The first attempt at gastric cancer typing was made by researchers in Singapore in 2013 8. Based on genomic expression, three main types were identified, namely proliferative, metabolic, and mesenchymal, while the 2014 Cancer Genome Atlas (TCGA) group classified gastric cancer into four subtypes based on six different molecular biology approaches 9: chromosomal instability (CIN), microsatellite instability (MSI), genome stability (GS), and EBV-positive (Epstein-Barr virus positive, EBV+) types.

### Genome based gastric cancer classification

TCGA is a landmark study that combines data from different platforms to comprehensively report the genetic variability associated with gastric cancer, and fully demonstrates the unique genomic characteristics of each molecular subtype, opening a new era of molecular typing of gastric cancer. This kind of molecular data spectrum-based typing can provide more accurate efficacy and prediction information than the traditional tissue system classification, making it possible to provide precise targeted therapy for gastric cancer** (Table 1)**.

The Asian Cancer Research Group (ACRG) in 2015 also proposed a new molecular typing of gastric cancer as microsatellite stable/epithelial-mesenchymal transformation (MSS/EMT) type, microsatellite unstable (MSI) type, microsatellite stable/tumor protein 53 active (MSS/TP53+) type and microsatellite stable/tumor protein 53 inactive (MSS/TP53-) type 10.

## Precision surgical treatment of gastric cancer

Among all the clinical treatments currently available for gastric cancer patients, surgery is the only treatment that can completely eradicate gastric cancer, and is also the foundation of gastric cancer treatment 2, 3. The ever-changing surgical methods and techniques of gastric cancer surgery are still somewhat controversial, but with the participation of more and more experts and scholars and the publication of authoritative research results, the hot topics of discussion are gradually reaching a unified consensus on a global scale** (Figure 1).**

As a class of highly heterogeneous tumors, different subtypes often show differentiated biological behaviors. The proliferation and metastasis of gastric cancer involve many molecular pathways: heterogeneity in cell proliferation, vascular metastasis in metastasis, and EMT pathway. With the development of molecular biology technology, we provide molecular typing and individualized molecular and immunotherapy for gastric cancer. Resectable gastric cancer preoperative refined imaging evaluation and artificial intelligence pathological diagnosis application; minimally invasive and surgical robots, precise operation of intraoperative fluorescent navigation and sophisticated perioperative management guided by the concept of accelerated rehabilitation is the direction of gastric cancer treatment in era of precision medicine.

### Application of precise preoperative assessment of resectable gastric cancer

Accurate preoperative staging and resectability assessment of gastric cancer is the key to successful surgery. Traditional gastrointestinal imaging, CT abdominal enhancement and general gastroscopy can clarify the nature and location of lesions and the presence of distant metastasis, but it is difficult to assess the depth of primary lesion infiltration (T stage) and the degree of regional lymph node metastasis (N stage) and evaluate the efficacy of neoadjuvant or translational therapy.

With the development of modern imaging and endoscopic equipment, including multislice spiral CT (MSCT), energy CT, MRI, PET-CT, and endoscopic ultrasound, pigmented endoscopy, and magnified endoscopy in the preoperative evaluation of gastric cancer, the accuracy of clinical staging of gastric cancer has been greatly improved 4, 11. Gastric MSCT combined with multiplanar reconstruction technique can not only accurately evaluate the lymph nodes and distant metastasis in the perigastric region, but also determine the anatomy and course of major blood vessels in the perigastric region, which brings great convenience to laparoscopic gastric cancer surgery. Spectral CT can improve the resolution of the tissue, and spectral curves and material separation techniques can quantitatively characterize the tissue composition of the extraplasmatic adipose tissue spaces and improve the accuracy of T-staging, especially T4a 12. *The problem of morphologic target lesions.* Among endoscopy methods, endoscopic ultrasound has the advantage of evaluating T stage and perigastric lymph node metastasis, while magnified endoscopy can accurately determine the extent and nature of early lesion involvement, playing an important role in endoscopic treatment and preoperative lesion localization 13.

### The development and trend of minimally invasive surgery of gastric cancer

With the development of minimally invasive technology and the introduction of precision medicine, the changes in the concept of gastric cancer surgery are mainly reflected in the following: on the basis of ensuring complete removal of tumor tissues, rational selection of surgical technique and scope of resection, preserving the function of the digestive tract as much as possible, reducing damage to the body and alleviating patients' pain. As medical technology continues to progress, it also pays more attention to the subjective needs of patients, hoping to cure the disease and minimize the trauma to the body at the same time 2, 14. As for the treatment of gastric cancer, minimally invasive surgery mainly includes laparoscopic surgery and da Vinci robotic system surgery. Among the minimally invasive surgeries, laparoscopic surgery has considerable unique advantages, but patients with advanced gastric cancer need to undergo lymph node dissection, which is more difficult and complicated, so laparoscopic surgery is only suitable for patients with early gastric cancer. However, experts and scholars still have great expectation for laparoscopic surgery to be applied to patients with advanced gastric cancer, and are in the process of further research.

In the 5^th^ edition of the Japanese guidelines for gastric cancer treatment published in 2018 15, laparoscopic surgery can be a routine option for distal gastrectomy for gastric cancer with clinical stage I. A phase II study in Japan included a study conducted in 2007. A phase II study in Japan included 176 patients from November 2007 to September 2008, of whom 140 cases were at pathological stage Ia, 23 cases were at stage Ib, 9 cases were at stage II, and 4 cases were at stage IIIa. 3 cases died during the follow-up period, and the 5-year survival rate was 98.2%, and the 5-year recurrence-free survival rate was 98.2%. This study showed that the long-term outcome of patients with stage I gastric cancer treated with laparoscopic gastrectomy was comparable to that of patients undergoing open surgery. Currently, there is a lack of evidence-based support for the feasibility of laparoscopic distal resection for gastric cancer with clinical stage II or higher 16-21.

The da Vinci robotic surgical system is capable of presenting a clear three-dimensional field of view, applying more flexible artificial joints and automatic tremor removal, etc. In 2000, the U.S. Food and Drug Administration (FDA) approved the da Vinci Surgical System (DVSS) for clinical use, but due to its expensive equipment and high technical requirements, the clinical rollout has been slow and robotic gastrectomy. The use of robotic gastrectomy (RG) for the treatment of gastric cancer has been studied for more than a decade, and there is a lack of evidence that it can be used as a standard of care 22-24. Gastric cancer surgeons expect the use of RG to overcome some of the disadvantages of conventional laparoscopic gastrectomy (LG) and to improve its safety, repeatability, and long-term outcome. However, the only large non-randomized prospective study (NCT 01309256) comparing DVSS with LG showed that the use of DVSS increased operative time and cost without reducing post-gastrectomy complications, suggesting that the full use of this surgical system in gastrectomy surgery remains controversial 24. How to minimize the cost and maximize the advantages of the system during treatment is still under further study.

### Discussion on the scope of lymph node dissection

Throughout the development of gastric cancer surgery, the scope of surgery for gastric cancer has gone through the process of “from small to large, and then from large to small”. In the early stage of understanding, how to completely remove the tumor has become a misunderstanding in clinical practice that the surgery is always “bigger”, and lymph node dissection has been carried out from D1 to D2, then from D2 to D3, and even combined with multi-organ resection 3, 5, 25.

The earliest definition of lymph nodes in gastric cancer was made by the Japanese Gastric Cancer Association, which was the first to define 3 stations of lymph nodes, and defined the corresponding surgical names according to the differences in the extent of lymph node dissection during surgery 25-27. In terms of the extent of lymph node dissection, D2 lymph node dissection has been recommended as a standard procedure in Japan since the 1960s, and Asian gastric cancer surgeons have routinely and routinely performed this procedure with good results 28. Asian gastric cancer surgeons have routinely and routinely performed this procedure with good results, whereas European and American surgeons often perform more limited lymph node dissection, which may result in inadequate tumor resection and is partly responsible for the difference in survival rates between the East and West.

As research progresses, in 2011, the Japanese Society of Gastric Cancer has redefined the scope of lymph node dissection in radical gastric cancer surgery, and believes that the scope of lymph node dissection should be determined according to the extent of gastrectomy, which improves the convenience of the surgery 29. Multiple single-center and multicenter data have shown that D2 lymph node dissection is the most effective surgical approach for advanced gastric cancer and gastric cancer with positive lymph node metastasis. When patients with progressive gastric cancer underwent D1 and D2 lymph node dissection, patients who underwent D2 lymph node dissection had a higher five-year survival rate. However, lymph node dissection that is more extensive than the D2 procedure does not significantly improve 5-year survival.

### Diagnosis of early gastric cancer

The development and evolution of the treatment of early gastric cancer (EGC) more fully embodies the concept of precision surgery. Along with the continuous improvement of endoscopic techniques and innovation of operating instruments and energy devices, endoscopic mucosal resection (EMR) and endoscopic submucosal dissection (ESD) have become the most popular methods for EGC treatment 30. It is an important tool for treatment. The latest Japanese JCOG0607 trial results show that for intestinal mucosal carcinoma without ulcers and with a diameter of >2 cm or with ulcers and with a diameter of ≤3 cm, i.e., EGC that meets the indications for ESD extension, ESD should be used as a standard treatment option instead of conventional surgical gastrectomy. *Adoption.* From surgical gastrectomy to precise endoscopic resection of lesions, the reduction in the scope of gastrectomy has brought about the preservation of complete gastric function. At the same time, based on the in-depth study of the characteristics of EGC lymph node metastasis, the emergence of sentinel node navigation surgery (SNNS) and other techniques has made it possible for the future EGC lymph node dissection to shift from regional dissection to more precise targeted dissection 20, 30. The standard approach to SNNS for gastric cancer is to perform precise perigastric lymph node dissection by preoperative or intraoperative injection of a tracer around the lesion to anticipate lymphatic areas that are likely to metastasize.

*About minimally invasive surgery for early gastric cancer.* With the continuous improvement in gastric cancer detection technology, more and more gastric cancer patients can be identified at an early stage. In order to maximize the quality of life of gastric cancer patients after surgery, whether patients with early gastric cancer need to undergo lymph node dissection at the beginning of diagnosis has become a hot topic of discussion. Related scholars have conducted linkage studies from other malignant tumor cases with the intention of determining whether sentinel lymph nodes exist in gastric cancer and whether lymph node metastasis in gastric cancer patients can be determined by detecting sentinel lymph nodes. If sentinel lymph nodes are present in gastric cancer patients, and then sentinel lymph node technology can be used in gastric cancer treatment, it will continue to promote the development of more individualized and precise treatment for early gastric cancer. Nanocarbon and ICG intraoperative lymph node visualization for precise dissection 30-33.

### Application of accelerated rehabilitation surgery in gastric cancer

Accelerated rehabilitation surgery is the use of a series of evidence-based perioperative optimization measures to reduce patient stress, promote rapid recovery, reduce patient length of stay, and reduce hospital costs. The concept of accelerated rehabilitation was elaborated by Danish scholar Kehlet in 1997 34, then developed and standardized in colorectal surgery, and has been steadily developing in orthopedics, cardiothoracic surgery, hepatobiliary surgery and other disciplines. Currently, the evidence base for perioperative accelerated rehabilitation surgery for gastric cancer gastrectomy is still inadequate, but the clinical practice is still actively expanding and exploring, and the first expert consensus on accelerated rehabilitation surgery for gastric cancer gastrectomy was published in China in 2017 35. In the 2018 update of the Chinese Expert Consensus on Accelerated Recovery Surgery and Pathway Management Guidelines, it is pointed out that the current consensus is mostly participated by surgeons, while the participation of anesthesia, nursing and other related specialties is relatively low, suggesting that the clinical community should strengthen the collection of more evidence-based medicine and further improve and revise the relevant measures under the model of multidisciplinary collaboration in the future 36, 37. Complications after gastric cancer surgery include infection, incision dehiscence, duodenal stump fistula, intestinal obstruction, anastomotic fistula, gastroparesis, impaired liver function, abdominal hemorrhage, thromboembolism, and anastomotic obstruction, the most common is infection among these 38. In recent years, with the advancement of medical technology, the incidence of postoperative complications in patients has decreased 39. This is because the improvement of gastric cancer surgery and the standardization of surgical cleaning range have made the survival rate of patients after surgery to a certain extent, the application of stapler shortens the operation time and reduces the risk of operation; In addition, there are perioperative nutritional supplements reduces the probability of certain complications. In order to reduce the occurrence of postoperative complications of gastric cancer, dealing with related complications before operation and shortening the operation time during the operation may play a certain preventive effect on the occurrence of postoperative complications. Through the application of new technologies such as artificial intelligence and big data, we can further optimize perioperative management, formulate corresponding preventive measures, and reduce the incidence of postoperative complications.

## Neoadjuvant treatment of gastric cancer

**Figure 2:**
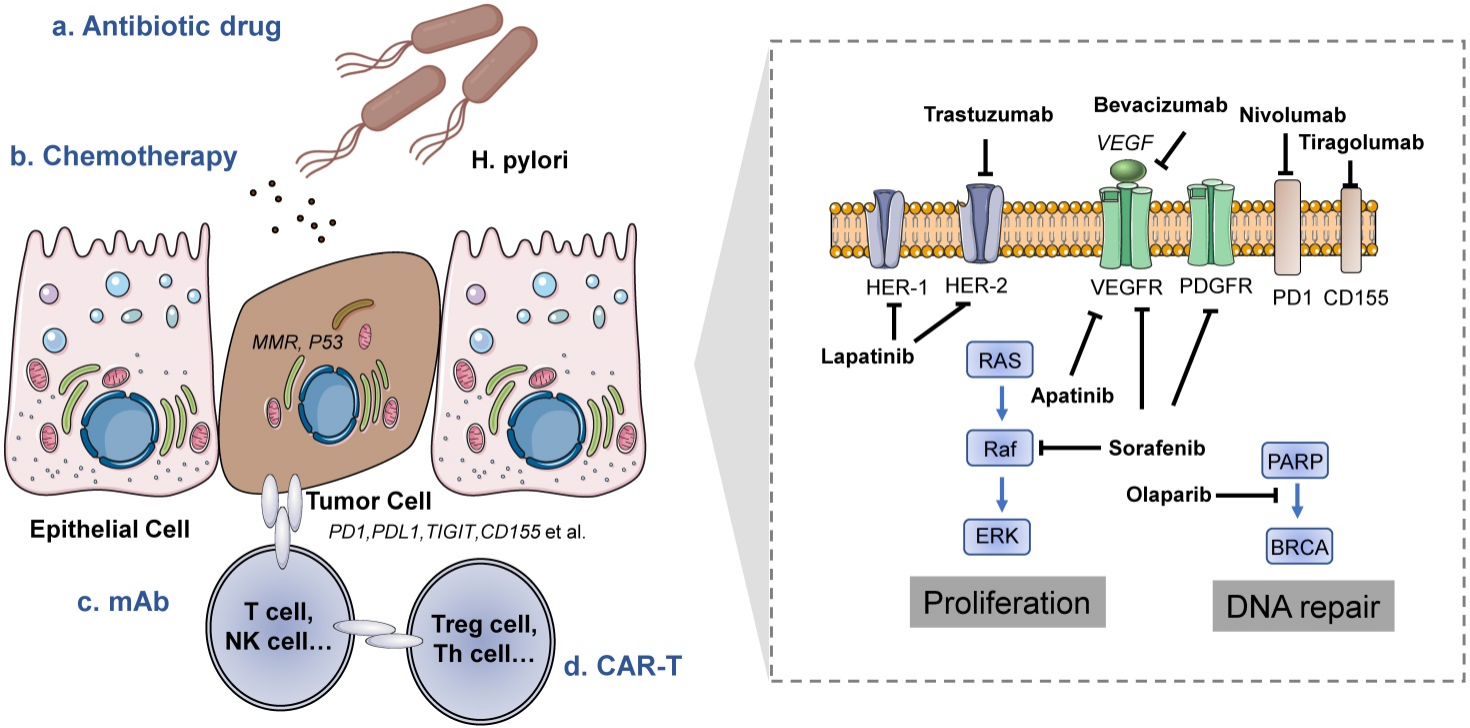
** Treatment methods of gastric cancer.** Left is the tumor microenvironment of gastric cancer and the target cells of different treatment methods. Right is a schematic diagram of the mechanism of drugs targeting gastric cancer cells.

For patients with gastric cancer, surgery is basically the only possibility of cure at this stage; however, since gastric cancer is insidious and lacks specific symptoms, most patients are already in advanced stages at the time of initial diagnosis, and adjuvant therapy improves the survival of operable gastric cancer patients 12. How to incorporate new treatment methods into the standard of care and thus improve the survival prognosis of these patients is an important issue in clinical research **(Figure 2).**

### Traditional chemotherapy

In 2017, a study by Al-Batran et al demonstrated that the use of a more effective chemotherapeutic agent in the chemotherapy regimen (FLOT regimen: docetaxel, 5-Fu, calcium folinic acid, and oxaliplatin) may improve patient outcomes, making FLOT the appropriate new standard of care for perioperative chemotherapy in patients with resectable gastric cancer, which is listed as a recommended regimen in international guidelines 29, 40-43.

### Potential molecular therapeutic targets for gastric cancer

Gastric cancer has a high degree of intra- and inter-tumor heterogeneity, and at the same time, intra-tumor heterogeneity has both temporal and spatial heterogeneity, which leads to a diversity of molecular variants, so precise molecular typing is a prerequisite for realizing molecularly targeted drug therapy 7, 44, 45. Several promising therapeutic targets in the field of gastric cancer are briefly summarized.

#### Anti-HER2 therapy

The ToGA study established the role of trastuzumab in the first-line treatment of patients with *HER2*-positive advanced gastric cancer and also established the star role of *HER2* in gastric cancer, although subsequent studies of several targeted* HER2* agents such as patumumumab, lapatinib, and T-DM1 in gastric cancer have all failed 46-49, but the development of drugs targeting *HER2* has not stopped.

#### Anti-angiogenic Therapy

The first antiangiogenic drug evaluated in gastric cancer, bevacizumab, failed, but other drugs in this class still show promise in gastric cancer and exploration continues. Apatinib is a small-molecule multi-targeted tyrosine kinase inhibitor developed in China, and its targets include *VEGFR-2*, *c-kit*, etc. Apatinib can significantly prolong the survival of patients with advanced gastric cancer when used in third-line or higher therapy, and based on this, CFDA has approved the use of apatinib for gastric cancer indication 50, 51.

#### PARP inhibitors, MET inhibitors

The phase III GOLD study in gastric cancer failed because the endpoint was not reached, but the subgroup analysis showed that olaparib could significantly benefit some patients and has some therapeutic potential in gastric cancer, but it is necessary to define the benefit groups, platinum-sensitive tumors and DNA 52. *MET* is a transmembrane tyrosine kinase receptor that binds to *HGF* and triggers a downstream cascade reaction. Both antibodies targeting *MET* and small-molecule inhibitors have failed in studies of *MET*-positive gastric cancer, and the lack of uniform standards for detecting *MET* expression in gastric cancer has resulted in different rates of positive *MET* expression in different studies 53.

### Molecularly Targeted Therapy and Drug Resistance

Targeted therapy in gastric cancer is relatively backward, and trastuzumab remains the only first-line targeted therapy, while *HER2* is the star molecule in gastric cancer. Since trastuzumab was first approved for use in breast cancer studies, most of the trastuzumab resistance mechanism studies have come from breast cancer studies and a few from gastric cancer studies. There is evidence that membrane receptors other than *HER2* can cause secondary resistance to targeted *HER2* therapy, such as overexpression of *MET* and its ligand *HGF* decreases trastuzumab sensitivity, upregulation of the membrane receptor *IGF-1R* is also associated with trastuzumab resistance, upregulation of *EphA2* expression triggers trastuzumab resistance, and activation of *HER4* by its ligand *NRG1* is involved in trastuzumab resistance. Downstream of* HER2, PI3K/AKT* pathway activation causes secondary drug resistance.

### Immunotherapy

In recent years, the study of the immune microenvironment has become a hotspot, especially the relationship between tumor immune escape and the surrounding immune environment has shifted from basic to clinical, and immunotherapy represented by PD-1/PD-L1 inhibitors has achieved breakthroughs in the treatment of many tumors 54, 55. Combined with the results of multiple clinical trials that have been conducted so far, PD-L1 expression as a biomarker for gastric cancer immunotherapy has drawn mixed conclusions. In 2017, the anti-PD-1 antibodies pembrolizumab and nivolumab were approved in the U.S. and Japan, respectively, for the treatment of patients with chemotherapy-resistant gastric cancer. However, the efficiency of anti-PD-1 antibody monotherapy in patients without PD-L1 screening was less than 12%, so monotherapy with anti-PD-1 cannot be a necessary treatment for patients with operable gastric cancer 3, 12, 56. In order to optimize the efficacy of immunotherapy, we still need to explore the combined immunotherapy strategies, such as immunotherapy combined with immunotherapy, immunotherapy combined with targeted therapy, immunotherapy combined with chemotherapy, etc., which are also the hotspots and directions for the investigation of gastric cancer immunotherapy.

Currently, there is no gold standard therapeutic adjunct, but neoadjuvant chemoradiotherapy plus surgery plus adjuvant chemoradiotherapy can be applied to the appropriate population. In the future, risk stratification based on PET, R1 status, lymph node metastasis, and microsatellite instability (MSI) may help to individualize treatment choices for the greatest benefit of the patient 56.

## Precise gastric cancer surgical based on artificial intelligence and big data

The earliest work on artificial intelligence (AI) in medicine dates back to 1970, and the broad concept of AI was first published at a Dartmouth College conference in 1956. As a part of artificial neural networks, deep learning is one of the rapidly growing fields in artificial intelligence, but its practical utility in the real world will depend on joint applications in multiple environments that allow the integration of medical knowledge-based tools with other applications, including medical record systems, outcome reporting systems, electronic prescribing systems, and, in the biological context, tools for genomic/proteomic data management and analysis. Artificial intelligence has been increasingly involved in disease diagnosis, treatment, and drug development, etc. 4, 14. Kanesaka et al reported a computer-aided system for identifying early gastric cancer with good diagnostic performance (96.3% accuracy), indicating the great potential of computer-aided diagnosis of early gastric cancer, especially in countries with a high incidence of gastric cancer but a low detection rate of early gastric cancer 11. More than 600,000 gastric cancer patients are diagnosed every year in China, and more than 80% of them are in advanced stage with poor prognosis. Computer-aided methods are expected to play an important role in the detection of early gastric cancer. Pathology is the current hotspot of artificial intelligence applications. Based on the unification of deep learning, statistics and informatics, AI is mainly applied in the field of gastric cancer surgery in the diagnosis of benign and malignant tumor pathology, staining analysis, early screening of cancer and the extensive development of molecular targeted drugs, etc. Automatic image analysis is a research hotspot in the field of surgical pathology.

In the era of artificial intelligence and big data, the application of precision medicine in gastric cancer surgery is still based on standardized diagnosis and treatment. Artificial intelligence and big data are indispensable in the data collection, processing and application of precision medicine in gastric cancer surgery, such as data processing of cases and case specimens, interpretation of heterogeneous data and specific treatment of specific cases. Based on the clinical data of more than 10,000 cases in the international arena, the analysis of the above data has led to the emergence of norms that are applicable to the treatment in China and even internationally 57. Although many medical centers in China have established information systems, these systems lack unified standards, so studies based on these data have problems such as small study samples, different treatment strategies, etc., which lack universality and waste resources to some extent. Through the construction of the big data research platform of the Gastrointestinal Oncology Union, it is hoped that in the subsequent development process, the standardization of “data” and broader and deeper communication will promote the further standardization of diagnosis and treatment, as well as the improvement of data quality, thus forming a virtuous cycle.

Many medical behaviors require the emotional participation of doctors; it is difficult for artificial intelligence to transmit the temperature of the doctor's face-to-face consultation in a short period of time for intelligent medical treatment. Establishment of large databases involves amount of important information, the protection of patient privacy faces unprecedented threats and challenges.

## Conclusion

In the era of precision medicine, along with the rise of artificial intelligence and big data, the diagnosis and treatment strategies of gastric cancer surgery have become more and more refined. Through accurate assessment of preoperative gastric cancer type and stage, precise, standardized, minimally invasive and individualized surgical treatments should be chosen. For patients with intermediate and advanced gastric cancer, interventions such as targeted therapy or immunotherapy combined with molecular targets are the key steps to realize the precision surgical treatment of gastric cancer. The author believes that in the near future, on the basis of integrating individual genomic information of gastric cancer patients with clinical, imaging and pathological data, the multidisciplinary diagnosis and treatment team of gastric cancer will be able to better realize individualized interpretation of the disease and provide patients with tailor-made and comprehensive treatment, which will truly reflect the precision of gastric cancer treatment.

## Figures and Tables

**Table 1:** Overview of gastric cancer TCGA classification

Subtype	Characteristics	Treatment
Microsatellite instability, MSI	Accounts for 22%. It is more common in gastric antrum or pylorus, especially in women; high mutations in genes encoding cancer signal proteins: PIK3CA; MHL1 promoter hypermethylation, gastric CIMP	Methylation inhibitor
Chromosomal instability, CIN	Accounts for 50%. It occurs frequently at the gastroesophageal junction and cardia, mostly intestinal type; TP53 mutations are significantly aneuploidy and RTKs genes are frequently amplified, such as: ERBB2, EGFR, ERBB3, VEGFR, PDGFR, FGFR, etc.	For RTKs; anti-angiogenic therapy
Genomically stable, GS	Accounts for 20%, mostly diffuse type. CDH1 mutation, RHOA gene mutation or RHO family GTPase activation protein gene fusion is more common	For RHOA
EBV positive	Accounted for 9%, PI3KCA high frequency mutation, P16 inactivation, PD-L1/L2 expansion	PI3K, CDK4/6 inhibitor; immunotherapy

## Abbreviations

TCGA: The Cancer Genome Atlas
ACRG: Asian Cancer Research Group
CIN: chromosomal instability
MSI: microsatellite instability
GS: genome stability
EBV-positive: Epstein-Barr virus positive
HER2: human epidermal growth factor receptor 2
TP53: tumor protein p53
PARPi: Poly (ADP-ribose) polymerase inhibitor
RG: robotic gastrectomy
LG: conventional laparoscopic gastrectomy
EGC: early gastric cancer
EMR: endoscopic mucosal resection
ESD: endoscopic submucosal dissection
SNNS: sentinel node navigation surgery
PD-1: Programmed cell death protein 1
AI: artificial intelligence
MSCT: multislice spiral CT
